# Epigenetic chromatin modifications in barley after mutagenic treatment

**DOI:** 10.1007/s13353-014-0226-9

**Published:** 2014-06-18

**Authors:** Agnieszka Braszewska-Zalewska, Marta Tylikowska, Jolanta Kwasniewska, Joanna Szymanowska-Pulka

**Affiliations:** 1 Department of Plant Anatomy and Cytology, University of Silesia in Katowice, Jagiellońska 28, Katowice, 40-032 Poland; 2 Department of Biophysics and Plant Morphogenesis, University of Silesia in Katowice, Jagiellońska 28, Katowice, 40-032 Poland

**Keywords:** Barley, Epigenetic modifications, Gamma ray, Maleic acid hydrazide, Immunostaining, Image cytometry

## Abstract

In addition to their normal developmental processes, plants have evolved complex genetic and epigenetic regulatory mechanisms to cope with various environmental stresses. It has been shown that both DNA methylation and histone modifications are involved in DNA damage response to various types of stresses. In this study, we focused on the involvement of two mutagenic agents, chemical (maleic acid hydrazide; MH) and physical (gamma rays), on the global epigenetic modifications of chromatin in barley. Our results indicate that both mutagens strongly influence the level of histone methylation and acetylation. Moreover, we found that gamma irradiation, in contrast to MH, has a more robust influence on the DNA methylation level. This is the first study that brings together mutagenic treatment along with its impact at the level of epigenetic modifications examined using the immunohistochemical method.

**Electronic supplementary material:**

The online version of this article (doi:10.1007/s13353-014-0226-9) contains supplementary material, which is available to authorized users.

## Introduction

Epigenetic chromatin modifications refer to heritable changes in gene expression that are not caused by changes in the nucleotide sequence of DNA. These chromatin modifications mainly concern DNA methylation and modifications of covalent histone N-terminal tails. Among them, methylated histone H3 at lysine 4 (H3K4), histone H3 at lysine 36 (H3K36) and acetylated histones H4 and H3, which are typical for euchromatin, methylated H3K9, H3K27, H4K20 and methylated DNA, which is typical for constitutive heterochromatin, are the ones that are examined most often. Epigenetic modifications in plants can be altered during the cell cycle (Jasencakova et al. 2001; 2003), plant development (Santamaría et al. 2009; Meijón et al. 2010) or in stress response (Luo et al. 2012).

A number of studies have shown that DNA and histone modifications play a key role in gene expression and development under stress in plants. Most of these stress-induced modifications are reset to the basal level once the stress is relieved, while some of the modifications may be stable and carried forward as a ‘stress memory’ (Chinnusamy and Zhu 2009). One of the aspects of stress-induced epigenetic modifications, which are not well recognised, is the changes that are induced by mutagens. It is known that mutagens affect processes like DNA replication or DNA repair, wherein epigenetic modification of chromatin are involved (Méchali et al. 2013). The involvement of epigenetic modifications in DNA repair pathways was identified in plants for double-strand breaks (DSB). Histone H2A variant phosphorylation (γH2AX) on the induction of DNA DSB using ionising radiation is an example of epigenetic modification involvement in repair pathways (Rybaczek and Maszewski 2007). Chromatin remodelling was also implicated in maize cells in response to UV-B. Acetylation at the N-terminal tails of histones H3 and H4 was increased in UV-B-treated plants (Casati et al. 2008).

The aim of the present study is to describe alterations in the level of global epigenetic modifications after two model mutagens that are commonly applied in plant mutagenesis, maleic hydrazide (MH) and gamma rays. These mutagens, especially gamma rays, are routinely used in plant mutagenesis and many new plant mutant varieties, including barley, have been developed through their application (Hagberg and Persson 1968; Schulte et al. 2009). However, the impact of these mutagens on epigenetic modifications has not been studied in plants to date.

MH, which is chemically defined as 1,2-dihydro-3,6-pyridazinedione, is a structural isomer of uracil. It is a clastogenic agent that acts in the S phase of the cell cycle and can lead to chromosome breaks, and it can also cause spindle fibre defects (Maluszynska and Maluszynski 1983). Although the mechanism of MH is not well known, there are some reports on its action as an inhibitor of the synthesis of nucleic acids and proteins, and also as a regulator of auxin metabolism (Ito et al. 2001). The mode of action of MH is possibly by its interference with the synthesis of uracil or becoming incorporated into RNA molecules replacing the uracil. The final result is the weakness in the structure of the chromosomes, leading to the chromosome breakage. The effect of MH on the proteins could influence the organisation of the spindle apparatus, which leads to the mitosis inhibition (Kaymak 2005).

Gamma rays, which are an ionising irradiation, produce reactive oxygen species (ROS), which interact with DNA and cause oxidative damage, such as base modification and single-/double-strand breaks (Morita et al. 2009). A detailed evaluation of the cytogenetic effects of MH and gamma rays in barley was previously done by our group (Juchimiuk et al. 2007; Juchimiuk-Kwasniewska et al. 2011).

In this study, the global nuclear level of different modifications at a microscopic level in barley were investigated using the immunostaining and image cytometry methods. We analysed the levels of H3K9me2 and DNA methylation (heterochromatin markers), as well as H4K5ac (a euchromatin marker), in *Hordeum vulgare* cells after treatment with mutagens. The analyses were performed following different post-treatment recovery times.

## Materials and methods

### Plant material and mutagenic treatment

Barley (*Hordeum vulgare*, 2n = 14) seeds of the ‘Start’ variety were used in the study. Two mutagens were used for the mutagenic treatment: MH and gamma rays. The mutagens doses used in this study were as applied in previous experiments on analyses of chromosome aberrations (Juchimiuk et al. 2007; Juchimiuk-Kwasniewska et al. 2011). Prior to the chemical treatment, the seeds were pre-soaked in distilled water for 8 h and then germinated in Petri dishes at 21 ºC in the dark for 40 h. The seedlings were treated with MH (Sigma, CAS 123–3301)  1, 2 or 3 mM for 3 h. After treatment, the seedlings were washed three times in distilled water. An analysis of the mitotic index and the frequency of micronuclei in the root cells were carried out immediately after treatment (0 h) and at 24 h post-incubation. The irradiation of seeds with 225 Gy of gamma rays was carried out at the International Atomic Energy Agency (IAEA), Seibersdorf Laboratory, Austria. After irradiation, the seeds were pre-soaked in distilled water for 8 h and germinated in Petri dishes at 21 ºC in the dark for 48 and 72 h. Control and treated material was fixed in ethanolglacial acetic acid (3:1) for chromosome aberration, mitotic activity and DNA methylation analyses. The material for the immunodetection of histones modification was fixed in 4 % formaldehyde. For mitotic activity and the frequency of micronuclei analyses, cytogenetic slides were prepared using the Feulgen squash technique. For each treatment and the control group, mitotic activity and the frequency of micronuclei were counted in 2,000 cells on each slide. Five slides, each made from two root meristems, were analysed for each experimental group.

### Immunostaining

The immunostaining was done for 3 mM of MH-treated plants and for 225 Gy of gamma ray-treated plants. The immunostaining was carried out as previously described (Braszewska-Zalewska et al. 2010, 2012, 2013). Briefly, the following rabbit monoclonal and polyclonal antibodies against modified histones and DNA were used: anti-acetyl histone H4 at lysine 5 (1:100; Millipore, cat. no. 04-118), anti-dimethyl histone H3 at lysine 9 (1:100; Upstate, cat. nos. 05-768 and 07-212), anti-5-methyl-cytosine (1:300, Abcam, cat. no. ab73938). Two secondary antibodies, Alexa Fluor 488 goat anti-rabbit IgG (Invitrogen, Molecular Probes, cat. no. A-11008) and Alexa Fluor 488 goat anti-mouse IgG (Invitrogen, Molecular Probes, cat. no. A-11001), were applied.

### Image acquisition and processing

The quantitative acquisition and analysis were performed using a high-content screening system (Scan^R, Olympus) based on a wide-field microscope Olympus IX81 equipped with a CCD camera ORCA-ER (Hamamatsu Photonics) and an MT20 illumination system based on a 150-W xenon mercury lamp. The automated segmentation of nuclei was based on threshold values (a border value of the fluorescence intensity of pixels between the background and the object). The analysis was performed assuming the following parameters of fluorescence intensities: ‘total’ (the sum of the pixel intensity value specific for the object) and ‘mean’ (the total intensity divided by the area of the object). The levels of epigenetic modifications were measured as an average value from the total Alexa 488 fluorescence intensities, which were carried out on at least 1,000 nuclei. The segmentation of the nuclei into the G1 and G2 phases was done based on total DAPI fluorescence intensities. Three independent acquisitions, each made from one meristem, for every epigenetic modification, were done. The difference in the level of a particular modification was deemed to be significant when at least a two-fold change in relation to the control was measured. Image processing operations were done using ImageJ 1.41 (Wayne Rasband, National Institutes of Health, USA).

### Statistical analysis

Normality of the signal intensity was assessed for each analysed group (Chi-square goodness-of-fit test, *p* < 0.05). As all the samples were large (*N* = 323 to 8,870), the *t*-test for independent statistical samples was used to check significant differences between the control and the treated samples.

## Results

### Analysis of mitotic activity and the frequencies of micronuclei

**Fig. 1:**
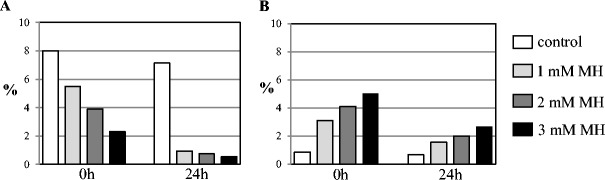
**A** The mitotic activity and **B** the frequency of micronuclei in *Hordeum vulgare* root meristematic cells induced by treatment with maleic acid hydrazide (MH) at 0 and 24 h post-incubation

**Fig. 2:**
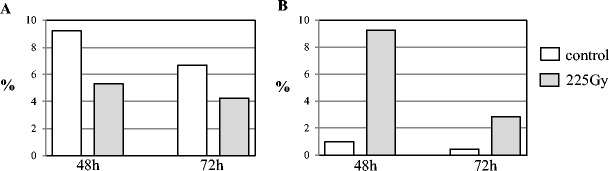
**A** The mitotic activity and **B** the frequency of micronuclei in *Hordeum vulgare* root meristematic cells induced by treatment with gamma rays after 48 and 72 h of germination

The mutagen doses applied in this study had been assessed as optimal for barley cells in our previous analyses (Juchimiuk et al. 2007; Juchimiuk-Kwasniewska et al. 2011). The mitotic activity and frequencies of micronuclei in barley root meristematic cells (control and treated with MH or gamma rays) were analysed in order to check whether the mutagenic effects under the applied treatment conditions are similar as in earlier studies. All MH concentrations reduced the mitotic activity of the barley cells (Fig. 1A). A lower mitotic activity was observed in cells that were treated with 3 mM MH. The frequency of micronuclei after treatment with MH varied from 1.5 to 5 %, depending on the concentration and post-incubation time (Fig. 1B). The highest frequency of cells with micronuclei was observed after treatment with 3 mM MH at 0 h post-incubation. A decrease in the frequency of micronuclei was observed when 24 h of post-incubation was applied for MH-treated roots. 3 mM MH has been chosen for the analysis of epigenetic modifications due to the strongest clastogenic effect, without total inhibitory effect. Gamma ray treatment also decreased the mitotic activity of the cells; however, the decrease was not as great as after MH treatment (Fig. 2A). 225 Gy of gamma ray induced micronuclei with a frequency of 2.8 % at 72 h and 9.3 % at 48 h of germination (Fig. 2B).

### Analysis of the epigenetic modifications

Three types of epigenetic modifications were analysed: H3K9me2, H4K5ac and 5mC. Prior to the analysis of the level of epigenetic modifications, the nuclei were segmented into G1 (2C DNA) and G2 (4C DNA) based on the total values of the DAPI fluorescence intensities (see Supplementary Data). The levels of epigenetic modifications were estimated based on the total values of the Alexa 488 fluorescence intensities which were measured for each pair of control and treated plants. Statistical analysis revealed that the majority of the samples demonstrated a non-normal distribution. In most cases, statistically significant differences in the signal intensity of the control samples and the treated samples were found (see Tables 1 and 2). It means that the mean values of these samples were different. However, in some cases, the medians of the same pairs of samples were similar, for example, in the case of H3K9me2 24 h G1 (see also Supplementary Data).

**Table 1:** Comparison of the mean Alexa 488 fluorescence intensity in the control (C) and maleic acid hydrazide (MH)-treated plants

		Mean*	SE*	Median*	No. of nuclei	*p*-Value**
H3K9me2 0 h						
G1	C	0.69	0.025	0.26	1,299	0.00
	MH	0.48	0.015	0.30	1,117	
G2	C	0.98	0.032	0.49	1,296	0.00
	MH	0.70	0.031	0.43	735	
H3K9me2 24 h						
G1	C	0.73	0.010	0.65	1,895	0.00
	MH	1.64	0.115	0.68	736	
G2	C	1.36	0.021	1.32	1,268	0.00
	MH	3.05	0.190	1.27	504	
H4K5ac 0 h						
G1	C	1.90	0.042	1.68	1,015	0.00
	MH	0.44	0.010	0.38	690	
G2	C	4.51	0.128	3.88	803	0.00
	MH	0.90	0.021	0.83	533	
H4K5ac 24 h						
G1	C	1.42	0.061	0.64	995	0.00
	MH	3.95	0.201	2.38	601	
G2	C	3.31	0.161	1.23	828	0.00
	MH	6.74	0.487	1.54	323	
5mC0 h						
G1	C	4.12	0.028	3.84	6,629	0.00
	MH	4.85	0.043	4.25	4,141	
G2	C	7.15	0.060	6.56	2,401	0.00
	MH	6.89	0.050	6.42	2,431	
5mC 24 h						
G1	C	8.06	0.063	7.70	5,018	0.53
	MH	8.14	1.077	7.10	1,978	
G2	C	10.4	0.211	6.40	1,916	0.27
	MH	10.8	0.188	9.76	819	

All data are presented in their relative units

*(×10^6^)

**Statistically significant differences on the basis of the *t*-test (*p* < 0.05)

**Table 2:** Comparison of the mean Alexa 488 fluorescence intensity in the control (C) and gamma ray (G)-treated plants

		Mean*	SE*	Median*	No. of nuclei	*p*-Value**
H3K9me2 48 h						
G1	C	0.48	0.007	0.38	1,773	0.00
	G	0.59	0.018	0.27	1,767	
G2	C	0.98	0.017	0.85	1,652	0.00
	G	1.29	0.039	0.67	1,398	
H3K9me2 72 h						
G1	C	0.24	0.005	0.18	1,378	0.00
	G	0.53	0.017	0.35	1,014	
G2	C	0.46	0.008	0.34	1,556	0.00
	G	0.85	0.023	0.61	1,258	
H4K5ac 48 h						
G1	C	2.74	0.059	1.96	2,483	0.94
	G	2.74	0.069	1.71	1,804	
G2	C	5.61	0.112	4.60	1,983	0.00
	G	7.41	0.191	4.76	1,479	
H4K5ac 72 h						
G1	C	1.58	0.025	1.09	3,724	0.67
	G	1.60	0.034	1.11	1,996	
G2	C	3.80	0.093	2.34	1,675	0.01
	G	3.50	0.072	2.52	1,998	
5mC 48 h						
G1	C	1.27	0.020	0.63	8,870	0.00
	G	1.99	0.039	1.27	2,495	
G2	C	2.34	0.050	1.17	4,690	0.00
	G	3.35	0.107	1.40	1,422	
5mC 72 h						
G1	C	1.29	0.017	0.82	6,265	0.00
	G	0.63	0.007	0.41	8,388	
G2	C	1.81	0.031	1.09	4,123	0.00
	G	1.06	0.015	0.78	3,637	

All data are presented in their relative units

*(×10^6^)

**Statistically significant differences on the basis of the *t*-test (*p* < 0.05)

For the MH treatment, a statistically significant difference was not indicated only for the 5mC 24 h samples, neither for G1 nor for G2. For the gamma ray treatment, a statistically significant difference was not detected in the case of H4K5ac 48 h for G1, as well as in the case of H4K5ac 72 h also for G1.

### MH treatment

**Fig. 3:**
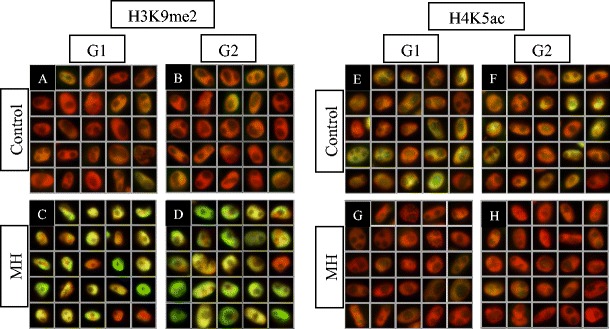
The level of H3K9me2 and H4K5ac after MH treatment. **A**–**D** H3K9me2, at 24 h post-incubation; **E**–**H** H4K5ac, at 0 h post-incubation; **A**, **E** control, G1 phase; **B**, **F** control, G2 phase; **C**, **G** MH, G1 phase; **D**, **H** MH, G2 phase; *red* DAPI staining (computer altered); *green* modified histones and DNA

The levels of epigenetic modifications were measured in the control and 3 mM MH-treated plants at two post-incubation times (0 and 24 h). The level of H3K9me2 at 0 h post-incubation was not strongly altered in MH-treated plants in comparison to the control; nevertheless, the samples differ statistically. However, at 24 h post-incubation, it was two–fold higher in MH-treated plants compared to the control (Fig. 3A–D; Table 1). In contrast to H3K9me2, the level of H4K5ac was four times lower in G1 and five times lower in G2 at 0 h post-incubation (Fig. 3E–H; Table 1). In turn, at 24 h post-incubation, this modification was almost three times higher in G1 and two times higher in G2 (Table 1). The level of 5mC was similar in the control and MH-treated plants at either 0 or 24 h post-incubation (Table 1). To summarise, the most relevant differences in the level of epigenetic modifications that were induced by MH treatment were detected for H4K5ac (see also Supplementary Data). After MH treatment, the levels of all the analysed modifications increased with the post-incubation times, while in the controls, they increased only for 5mC.

### Gamma ray treatment

**Fig. 4:**
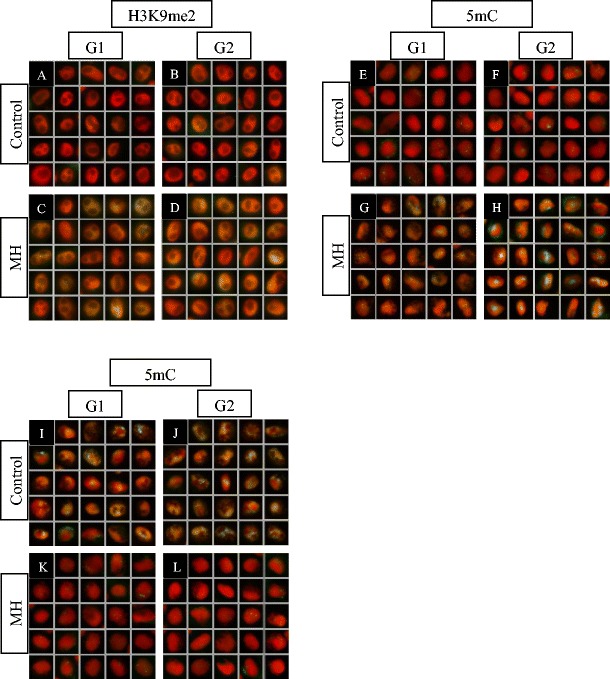
The level of H3K9me2 and 5mC after gamma ray treatment. **A**–**D** H3K9me2, after 72 h of germination; **E**–**H** 5mC, after 48 h of germination; **I**–**L** 5mC, after 72 h of germination; **A**, **E**, **I** control, G1 phase; **B**, **F**, **J** control, G2 phase; **C**, **G**, **K** gamma ray, G1 phase; **D**, **H**, **L** gamma ray, G2 phase; *red* DAPI staining (computer altered); *green* modified histones and DNA

The levels of epigenetic chromatin modifications were measured in the control and 225 Gy gamma ray-treated plants. After the seeds were irradiated, analyses in two periods of plants germination, 2-day-old seedlings (48 h of germination) and 3-day-old seedlings (72 h of germination), were carried out. The level of H3K9me2 after irradiation was similar in the 2-day-old seedlings in comparison with the control, but it was two-fold higher in G1 and almost two-fold higher in G2 in the 3-day-old seedlings compared to the control (Fig. 4A–D; Table 2). The level of H4K5ac in gamma ray-treated plants was not altered, especially in G1, in both 2- and 3-day-old seedlings (Table 2, see also Supplementary Data). The level of 5mC was more than one-fold higher in 2-day-old seedlings compared to the control (Fig. 4E–H; Table 2), contrary to the 3-day-old seedlings, where it was two-fold lower after treatment (Fig. 4I–L; Table 2). To summarise, the most relevant differences in the level of epigenetic modifications after gamma ray treatment were detected for DNA methylation. After gamma ray treatment, the levels of all the analysed modifications decreased with the post-incubation times, while in the controls, they increased with post-incubation times.

## Discussion

The frequencies of micronuclei in barley root meristematic cells, after MH and gamma ray treatment, were compared to the results with previously assessed mutagenic effects using the same mutagens doses. In present study, the highest frequency of cells with micronuclei was observed after seedlings treatment with 3 mM MH. An even higher frequency of micronuclei in barley cells induced by the same MH concentration, 3 mM, was shown by Juchimiuk et al. (2007). The differences in the mutagenic effects in root meristematic cells could be due to the stage of the plant development that was used for treatment: seeds, not seedlings, were used for treatments in previous experiments. Similarly, the previous study (Juchimiuk-Kwasniewska et al. 2011) showed a stronger clastogenic effect of 225 Gy of gamma ray in barley roots than did our analysis. However, in both experiments, the gamma rays were applied to seeds. The different frequencies of micronuclei in barley cells induced by the same dose of gamma rays can be explained by the other variations in the conditions of the experiments, such as germination times.

Chromatin remodelling through histone and DNA modifications plays an important role in the cellular response to DNA damage after stress conditions. Recent studies have highlighted the functional crosstalk between histone modifications and other proteins that are involved in DNA damage response (van Attikum and Gasser 2009). The involvement of epigenetic mechanisms in the response to environmental cues and to different types of abiotic stresses has been very well documented (Labra et al. 2002; Sokol et al. 2007; Kim et al. 2008; Sahu et al. 2013). Nevertheless, there are little data on the epigenetic modifications after ionising irradiation. Hyperacetylated histones H3 and H4 were present in maize at the promoter and transcribed regions of UV-B-regulated genes, but no changes in H3 methylation was detected (Casati et al. 2008). Additionally, enzymes that participate in DNA methylation were shown to be important during DNA repair after UV-B damage (Questa et al. 2013). In our study, a global decrease in H4 acetylation was detected in both MH- and gamma ray-treated plants in the first analysed post-incubation times. An overall decrease in H4 acetylation and an increase in H3 methylation may be linked to transposon repression. It was shown that HDA6 (a histone deacetylase) and MET1 (a histone methyltransferase) interact directly and act together to silence transposons by modulating DNA methylation, histone acetylation and histone methylation status in Arabidopsis (Liu et al. 2012).

The ability of epigenetic modifications to alter rapidly and reversibly could be a key component of the flexibility of plant responses to the environment. It was suggested that exposure to environmental stress could leave epigenetic marks in chromatin and keep the chromatin region in a ‘permissive’ state that may facilitate quicker and more potent responses to subsequent environmental changes, thus causing a transgenerational ‘memory’ (Luo et al. 2012; Mirbahai and Chipman 2014). The results presented in this paper indicate that epigenetic modifications are very dynamic phenomena. These changes could reflect the stress response of the cells after MH and gamma ray treatment, through chromatin remodelling. We also found that the mutagens used in this study have a distinct impact on histone and DNA epigenetic modifications. No obvious changes in the DNA methylation level were observed after MH treatment, while a significant increase and, later, a decrease in DNA methylation was detected after gamma ray treatment. It is possible that the development stage of plant material influenced the response to mutagens; in the case of gamma rays, seeds were treated, whereas seedlings were treated with MH. Changes in histone H3 and H4 modifications levels were observed after both mutagen treatments; however, they were more significant after MH. These may reflect distinct mechanisms involved in stress response after MH and gamma ray treatment. It is commonly known that the heterochromatin regions represent ‘hot spots’ of aberration formation induced by S-phase-dependent mutagens (Schubert et al. 1998, 2004). This hypothesis is consistent with the results of this study that show an increase in the level of H3K9me2, which is a heterochromatin-specific marker. The higher level of H3K9me2 after treatment with MH and gamma rays could be a response to DNA breaks, which has previously been confirmed for human cells (Altmeyer and Lukas 2013). On the other hand, an increase in the level of H4K5ac with the post-incubation times after MH treatment may be related to the relaxation of chromatin as a preparation for DNA repair and the activation of stress response genes. The data on the changes of histone acetylation pattern are only available for UV (Casati et al. 2008).

The response in the DNA methylation level depends on the stress factors (Labra et al. 2002; Kovalchuk et al. 2004). In this study, we showed that the changes in the DNA methylation level after gamma ray treatment were more dynamic than after MH treatment. It was shown for Arabidopsis that UV caused an increase in the level of methylation in the pericentromeric regions of chromosomes (Boyko et al. 2010); nevertheless, there are no similar studies investigating gamma rays or chemical mutagens.

## Conclusions

Our results indicate that epigenetic modifications are strongly affected after mutagenic treatment. Gamma rays caused more significant changes in the DNA methylation level, whereas maleic acid hydrazide (MH) caused more significant changes in the histones methylation and acetylation levels. This may imply that epigenetic modifications may be involved in specific aspects of the cellular answer to mutagenic treatment. These may encompass DNA damage repair, transposons silencing and the activation/repression of stress genes.

## Electronic supplementary material

Below is the link to the electronic supplementary material.

DAPI and Alexa 488 fluorescence intensity distributions in the G1 and G2 phases. (DOCX 1677 kb)
