# Supplementary material for: Epigenetic chromatin modifications in barley after mutagenic treatment
Source: J Appl Genet. 2014 Jun 18;55(4):449–56. doi: 10.1007/s13353-014-0226-9 (PMC4185110; doi:10.1007/s13353-014-0226-9)
Supplement: Supplementary file 1 — DAPI and Alexa 488 fluorescence intensity distributions in the G1 and G2 phases. (DOCX 1677 kb) [file 13353_2014_226_MOESM1_ESM.docx]

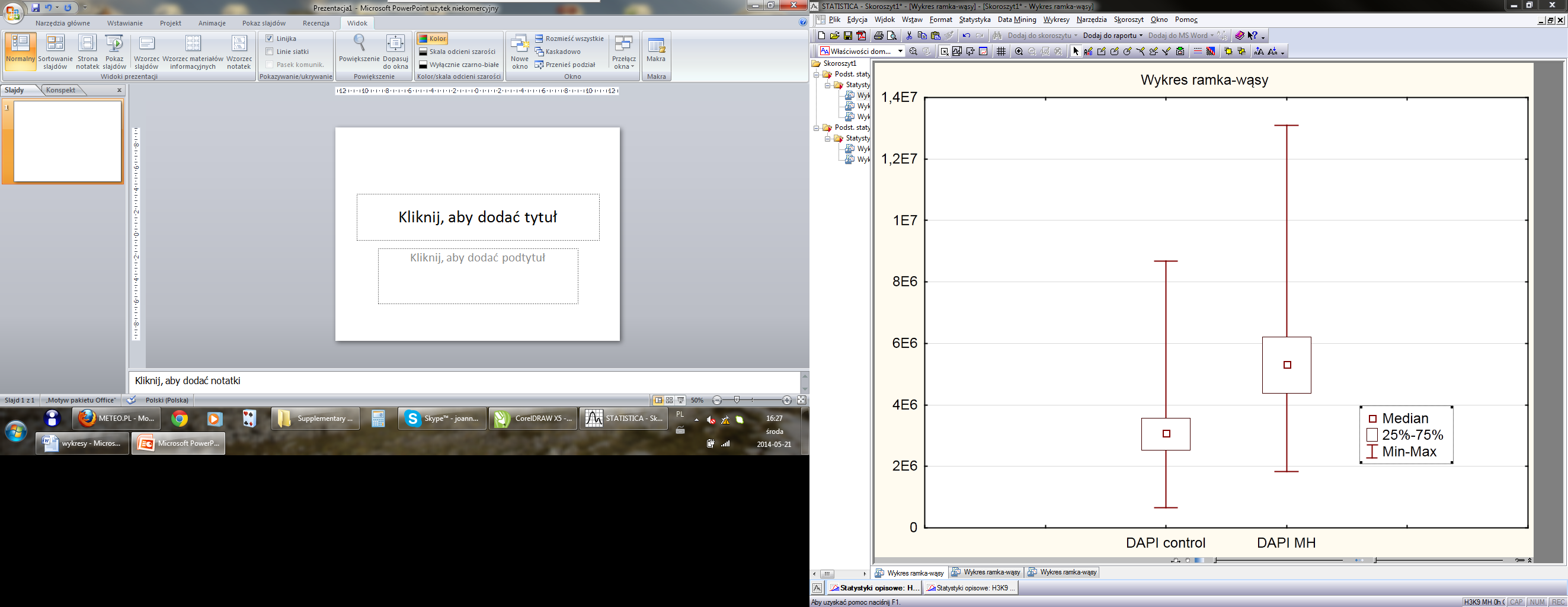


MH_3mM H3K9me2 0h

MH_3mM H3K9me2 MH 24h

MH_3mM H4K5ac_0h

MH_3mM H4K5ac_24h

MH_3mM 5mC_0h

MH_3mM 5mC_24h

gamma H3K9me2_48h

gamma H3K9me2_72h

gamma H4K5ac_48h

gamma H4K5ac_72h

gamma 5mC_48h

gamma 5mC_72h
